# Supplementary material for: Discovery of SNP Molecular Markers and Candidate Genes Associated with Sacbrood Virus Resistance in Apis cerana cerana Larvae by Whole-Genome Resequencing
Source: Int J Mol Sci. 2023 Mar 25;24(7):6238. doi: 10.3390/ijms24076238 (PMC10094193; doi:10.3390/ijms24076238)
Supplement: Supplementary file 1 [file ijms-24-06238-s001.zip › Figure S1 Verification and quantification of purified SBV..pdf]

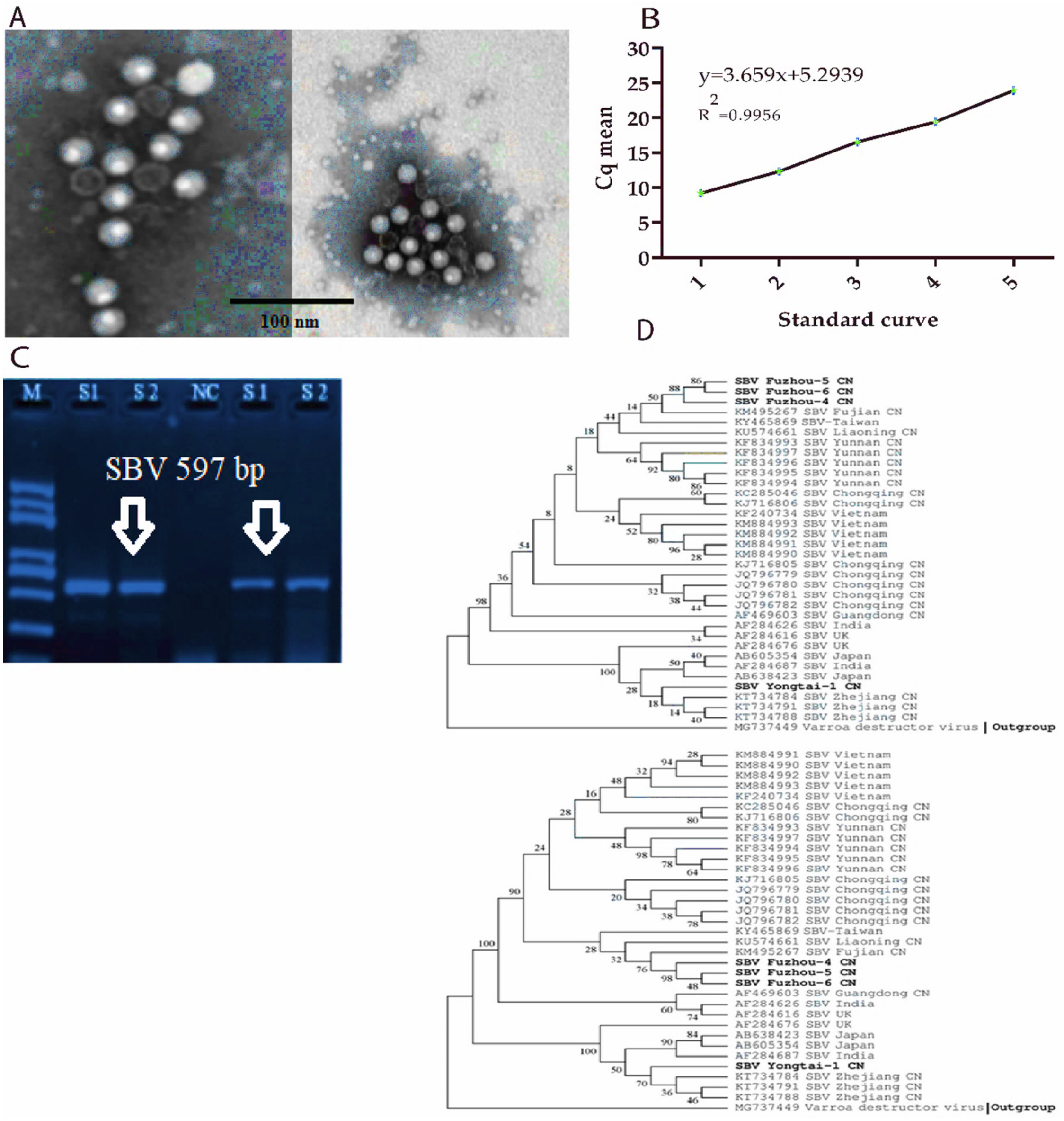

Figure S1 Verification and quantification of purified SBV.

(**A**) Observation of SBV particles under transmission electron microscope (TEM) SBV particles was imaged using HV= 80.0 KV, direct mag= 40,000x, print mag= 145,000 x, and ATM Camera system; (**B**) Standard curve of absolute qPCR from purified SBV sample result; (**C**) Detection SBV from purified samples on a 2% agarose gel electrophoresis in purified samples of larvae *A. c. cerana*, using Trans 2K plus DNA marker S1, sample 1; S2, sample 2 and NC, negative control and (**D**); Phylogenetic analysis of purified SBV of PCRs product using from Fuzhou and Yongtai and the other sequences were downloaded from NCBI and *varroa destructor* virus was used as control out of the group. Phylogenetic tree neighbor-joining and phylogenetic tree maximum likelihood were constructed using MEGA 11 with a bootstrap test of 1000 replicates.
