# Supplementary material for: Discovery of SNP Molecular Markers and Candidate Genes Associated with Sacbrood Virus Resistance in Apis cerana cerana Larvae by Whole-Genome Resequencing
Source: Int J Mol Sci. 2023 Mar 25;24(7):6238. doi: 10.3390/ijms24076238 (PMC10094193; doi:10.3390/ijms24076238)
Supplement: Supplementary file 1 [file ijms-24-06238-s001.zip › Figure S3 SNP mass distribution map between the samples..pdf]

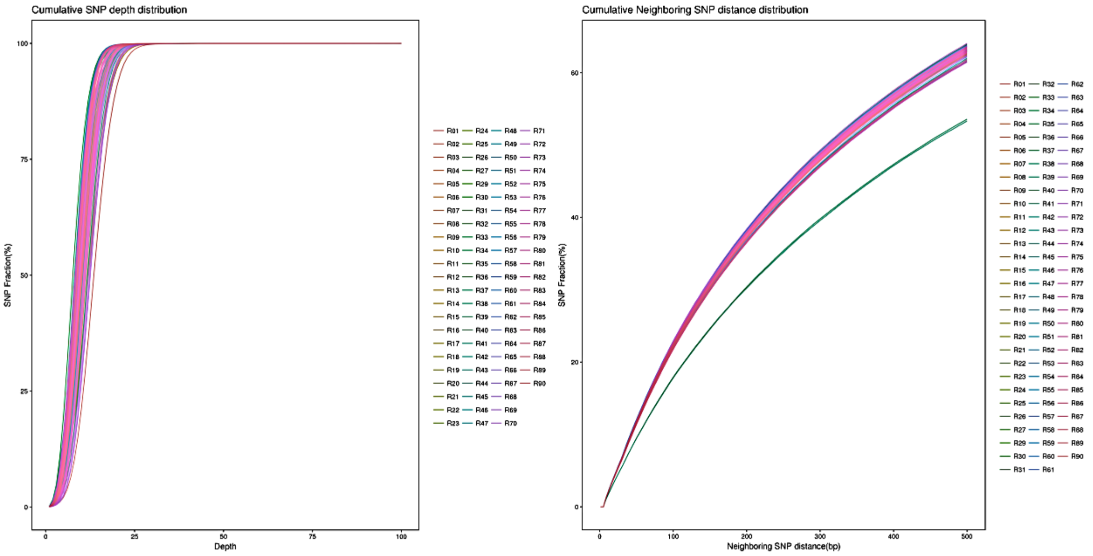

Figure S3 SNP mass distribution map between the samples.

In order to ensure the credibility of the sample SNPs, the number of reads supported for the SNPs detected on the sample and the cumulative statistical distribution of the distance of adjacent SNPs were conducted.

On the left is a cumulative plot of the supported SNP readings, and on the right is a cumulative plot of the distance between adjacent SNP R01 and R90, representing the consistency of the 90 accessions of *A. c. cerana*.
