# Supplementary material for: Discovery of SNP Molecular Markers and Candidate Genes Associated with Sacbrood Virus Resistance in Apis cerana cerana Larvae by Whole-Genome Resequencing
Source: Int J Mol Sci. 2023 Mar 25;24(7):6238. doi: 10.3390/ijms24076238 (PMC10094193; doi:10.3390/ijms24076238)
Supplement: Supplementary file 1 [file ijms-24-06238-s001.zip › Figure S4 The distribution of SNP mutations between samples..pdf]

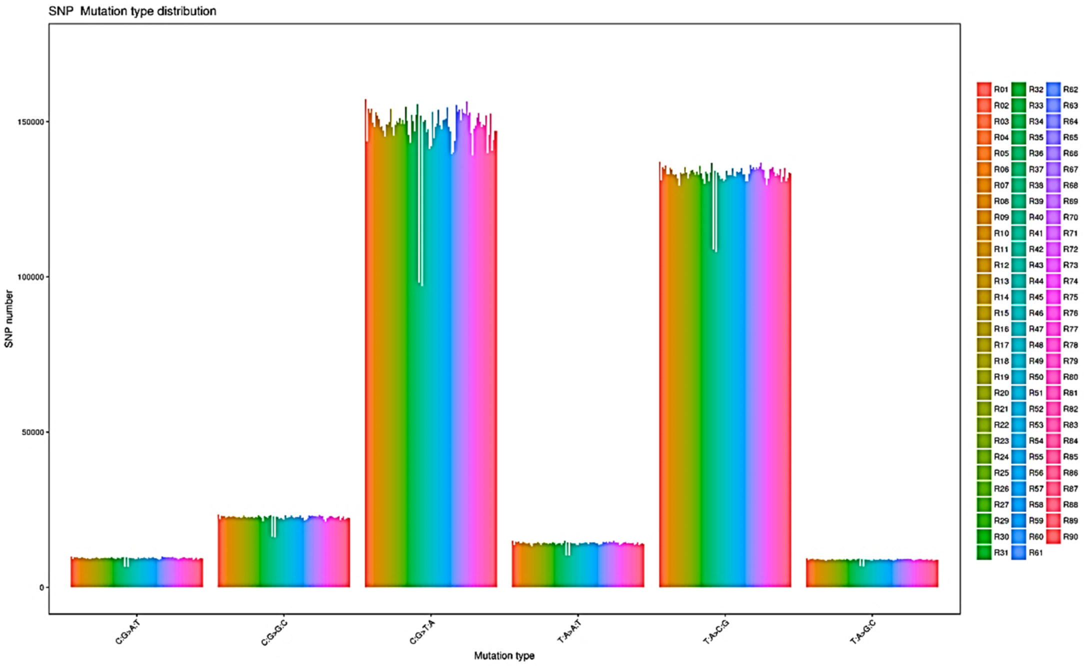

Figure S4 The distributions of SNP mutations between samples.

The vertical indicates the number of SNPs, and the horizontal indicates the mutation type of SNPs. Genome-wide SNP mutations can be divided into six categories. Taking T: A > C: G as an example, this type of SNP mutation includes T > C and A > G. When T > C mutations appear on the positive chain of the reference genome, the sequencing data are comparable to both the positive and negative chains of the reference genome, so the position of A > G mutations is the same as that of the negative chain of the reference genome, so T > C and A > G are classified into the same category.
