# Supplementary material for: Discovery of SNP Molecular Markers and Candidate Genes Associated with Sacbrood Virus Resistance in Apis cerana cerana Larvae by Whole-Genome Resequencing
Source: Int J Mol Sci. 2023 Mar 25;24(7):6238. doi: 10.3390/ijms24076238 (PMC10094193; doi:10.3390/ijms24076238)
Supplement: Supplementary file 1 [file ijms-24-06238-s001.zip › Figure S5 KEGG pathway enrichment analysis of target genes regulated by SNPs..pdf]

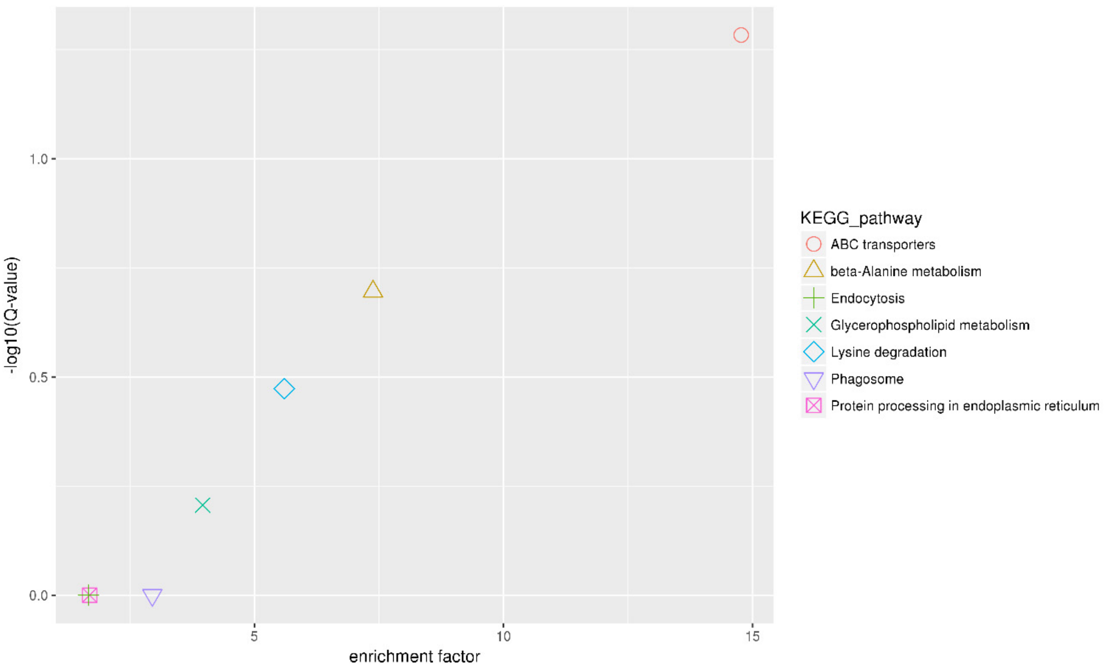

Figure S5 KEGG pathway enrichment analysis of target genes regulated by SNPs.

ApisCC1.0.int0.5maf0.05_FST_Pi_R_divides_S_0.95.KEGG result. Rich factor refers to the ratio of the number of genes located at this pathway in differentially expressed genes to the total number of genes located at this pathway in all genes. The larger Rich Factor is, the higher the enrichment degree. The shape indicates the genes enriched in which pathway.
