# Supplementary material for: Discovery of SNP Molecular Markers and Candidate Genes Associated with Sacbrood Virus Resistance in Apis cerana cerana Larvae by Whole-Genome Resequencing
Source: Int J Mol Sci. 2023 Mar 25;24(7):6238. doi: 10.3390/ijms24076238 (PMC10094193; doi:10.3390/ijms24076238)
Supplement: Supplementary file 1 [file ijms-24-06238-s001.zip › Figure S6 The frequency distribution of the Kinship value and linkage disequilibrium analysis..pdf]

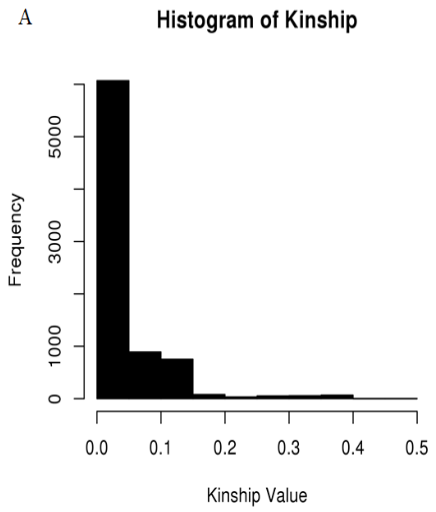

Figure S6 The frequency distribution of the Kinship value and linkage disequilibrium analysis.

(A) by using SPAGeDi software, it is possible to estimate the relative kinship between two individuals in a natural population. The affinity itself is a relative value that defines the genetic similarity between two specific materials and the genetic similarity between any materials, so when the result appears that the kinship value between the two materials is less than 0, it is directly defined as 0.
