# Supplementary material for: Discovery of SNP Molecular Markers and Candidate Genes Associated with Sacbrood Virus Resistance in Apis cerana cerana Larvae by Whole-Genome Resequencing
Source: Int J Mol Sci. 2023 Mar 25;24(7):6238. doi: 10.3390/ijms24076238 (PMC10094193; doi:10.3390/ijms24076238)
Supplement: Supplementary file 1 [file ijms-24-06238-s001.zip › Table S1 Samples information in detail..pdf]

Table S1 Samples information in detail.

No. of sample	Group	Phenotypic trait	Trait type	Sample type	Uniform sample ID
1	S_1_1	Susceptible	0	Larva	R01
2	S_1_2	Susceptible	0	Larva	R02
3	S_1_3	Susceptible	0	Larva	R03
4	S_1_9	Susceptible	0	Larva	R04
5	S_1_10	Susceptible	0	Larva	R05
6	R_1_1	Resistance	1	Pupa	R06
7	R_1_2	Resistance	1	Pupa	R07
8	R_1_3	Resistance	1	Pupa	R08
9	R_1_4	Resistance	1	Pupa	R09
10	R_1_6	Resistance	1	Pupa	R10
11	R_1_7	Resistance	1	Pupa	R11
12	R_1_9	Resistance	1	Pupa	R12
13	S_2_2	Susceptible	0	Larva	R13
14	S_2_3	Susceptible	0	Larva	R14
15	S_2_4	Susceptible	0	Larva	R15
16	S_2_5	Susceptible	0	Larva	R16
17	S_2_6	Susceptible	0	Larva	R17
18	S_2_7	Susceptible	0	Larva	R18
19	S_2_10	Susceptible	0	Larva	R19
20	S_2_13	Susceptible	0	Larva	R20
21	S_2_14	Susceptible	0	Larva	R21
22	R_2_1	Resistance	1	Pupa	R22
23	R_2_2	Resistance	1	Pupa	R23
24	R_2_3	Resistance	1	Pupa	R24
25	R_2_4	Resistance	1	Pupa	R25
26	R_2_5	Resistance	1	Pupa	R26
27	R_2_7	Resistance	1	Pupa	R27
28	R_2_11	Resistance	1	Pupa	R28
29	R_2_13	Resistance	1	Pupa	R29
30	R_2_14	Resistance	1	Pupa	R30
31	R_2_15	Resistance	1	Pupa	R31
32	S_3_1	Susceptible	0	Larva	R32
33	S_3_3	Susceptible	0	Larva	R33
34	S_3_4	Susceptible	0	Larva	R34
35	S_3_5	Susceptible	0	Larva	R35
36	S_3_6	Susceptible	0	Larva	R36
37	S_3_7	Susceptible	0	Larva	R37
38	S_3_10	Susceptible	0	Larva	R38
39	S_3_12	Susceptible	0	Larva	R39
40	S_3_13	Susceptible	0	Larva	R40
41	R_3_1	Resistance	1	Pupa	R41
42	R_3_2	Resistance	1	Pupa	R42
43	R_3_4	Resistance	1	Pupa	R43
44	R_3_5	Resistance	1	Pupa	R44
45	R_3_8	Resistance	1	Pupa	R45
46	R_3_9	Resistance	1	Pupa	R46
47	R_3_10	Resistance	1	Pupa	R47
48	R_3_13	Resistance	1	Pupa	R48
49	S_1_4	Susceptible	0	Larva	R49
50	S_1_5	Susceptible	0	Larva	R50

51	S_1_8	Susceptible	0	Larva	R51
52	R_1_5	Resistance	1	Pupa	R52
53	R_1_8	Resistance	1	Pupa	R53
54	R_1_10	Resistance	1	Pupa	R54
55	R_1_11	Resistance	1	Pupa	R55
56	R_1_12	Resistance	1	Pupa	R56
57	R_1_13	Resistance	1	Pupa	R57
58	R_1_14	Resistance	1	Pupa	R58
59	R_1_15	Resistance	1	Pupa	R59
60	S_2_9	Susceptible	0	Larva	R60
61	S_2_11	Susceptible	0	Larva	R61
62	S_2_15	Susceptible	0	Larva	R62
63	R_2_6	Resistance	1	Pupa	R63
64	R_2_8	Resistance	1	Pupa	R64
65	R_2_9	Resistance	1	Pupa	R65
66	R_2_12	Resistance	1	Pupa	R66
67	S_3_11	Susceptible	0	Larva	R67
68	S_3_14	Susceptible	0	Larva	R68
69	S_3_15	Susceptible	0	Larva	R69
70	R_3_3	Resistance	1	Pupa	R70
71	R_3_6	Resistance	1	Pupa	R71
72	R_3_12	Resistance	1	Pupa	R72
73	R_3_7	Resistance	1	Pupa	R73
74	R_3_11	Resistance	1	Pupa	R74
75	R_3_14	Resistance	1	Pupa	R75
76	R_3_15	Resistance	1	Pupa	R76
77	S_1_7	Susceptible	0	Larva	R77
78	S_1_6	Susceptible	0	Larva	R78
79	S_1_11	Susceptible	0	Larva	R79
80	S_1_12	Susceptible	0	Larva	R80
81	S_1_13	Susceptible	0	Larva	R81
82	S_1_14	Susceptible	0	Larva	R82
83	S_1_15	Susceptible	0	Larva	R83
84	S_2_1	Susceptible	0	Larva	R84
85	S_2_8	Susceptible	0	Larva	R85
86	S_2_12	Susceptible	0	Larva	R86
87	R_2_10	Resistance	1	Pupa	R87
88	S_3_2	Susceptible	0	Larva	R88
89	S_3_8	Susceptible	0	Larva	R89
90	S_3_9	Susceptible	0	Larva	R90
