# Supplementary material for: Discovery of SNP Molecular Markers and Candidate Genes Associated with Sacbrood Virus Resistance in Apis cerana cerana Larvae by Whole-Genome Resequencing
Source: Int J Mol Sci. 2023 Mar 25;24(7):6238. doi: 10.3390/ijms24076238 (PMC10094193; doi:10.3390/ijms24076238)
Supplement: Supplementary file 1 [file ijms-24-06238-s001.zip › Table S2 DNA concentration, DNA integrity quality Qubit integrity and OD ratio..pdf]

Table S2 DNA concentration, DNA integrity quality Qubit integrity, and OD ratio of 90 accessions of *Apis cerana cerana*.

Sample ID	Concentration	OD 260/288	OD 260/230	Qubit concentration(ng/uL)	volume (uL)	Total amount (ug)	Test result
R01	2375.3	1.93	2.01	166.4	37	6.2	A
R02	1773.6	2.1	1.93	108	37	8.6	A
R03	1887.8	2.04	2.13	306	37	3.5	A
R04	1903.3	2	1.89	193	37	2.2	A
R05	1355.7	2.07	1.89	166.4	37	6.2	A
R06	2050.6	2.13	2.08	125	37	2.2	A
R07	1728.8	2.12	2.19	136	45	6.1	A
R08	1890	2.04	2.11	100	44	4.4	A
R09	1728.8	2.12	2.19	136	45	6.1	A
R10	2919.7	1.84	1.26	162	23	3.7	A
R11	2488.7	2.01	2	138	44	6.2	A
R12	3062.3	2.13	2.23	40.2	37	1.5	A
R13	3062.3	2.13	2.23	84.9	37	3.1	A
R14	1056.5	2.03	2.22	264	45	11.9	A
R15	4288.7	2.13	2.21	165	45	7.4	A
R16	2348.8	2.06	2.09	40.2	37	1.5	A
R17	3066.6	2.11	2.03	84.9	37	3.1	A
R18	4361.8	1.82	1.22	306	20	6.1	A
R19	3785	2.09	2	264	45	11.9	A
R20	3383.4	1.93	1.85	436	44	19.2	A
R21	2856.6	2.13	2.14	133	38	5.1	A
R22	3478.7	1.82	1.28	346	45	6.9	A
R23	2628.1	1.92	2.06	266	44	11.7	A
R24	1953	2.08	2.05	296	37	5.9	A
R25	1491.1	1.99	2.03	50.2.0	60	3	A
R26	2535	1.82	1.95	264	45	11.9	A
R27	1373.3	2.08	1.99	46.4.0	60	2.8	A
R28	1889.1	2.1	2.07	43.2.0	60	2.6	A
R29	1529	2.11	2.14	40.4.0	60	2.4	A
R30	2207.8	2	2.07	226	47	10.6	A
R31	3785	2.09	2	151	45	6.8	A
R32	2231.1	2.11	2.09	120	45	5.6	A
R33	2265.7	2.15	2.26	181	45	8.1	A
R34	2139.2	2.16	2.3	151	45	6.8	A
R35	1368.1	1.98	1.92	144	28	4	A
R36	2039	2.16	2.27	125	42	6	A
R37	1279	1.97	1.91	194	32	6.2	A
R38	1884.5	1.97	1.83	97.4	47	4.6	A
R39	2904	2	1.96	125	45	5.6	A
R40	2156.7	2.07	2.27	181	45	8.1	A
R41	2317.1	2.02	1.99	85.2	47	4	A
R42	3157.3	2.08	1.98	76	37	2.8	A
R43	2148.3	1.76	1.67	244	45	6.3	A
R44	1151.6	1.98	1.98	162	32	5.2	A
R45	1917.2	1.98	1.91	71.4	47	3.4	A
R46	2036.5	2.05	1.94	160.9	37	6	A
R47	2157.7	1.97	1.83	79.6	37	2.9	A
R48	1268.7	1.98	1.96	232	32	7.4	A

R49	1903.3	1.67	1.67	312	32	10	A
R50	2416.3	2.02	2.05	220	28	6.2	A
R51	2203.7	2.05	2.21	53.88	60	3.2	A
R52	3322.7	2.02	1.92	306	37	6	A
R53	2642.5	2.02	2.1	67.4	60	4	A
R54	2740.9	2.01	1.98	65.88	60	3.9	A
R55	1553.3	2.11	2.09	79.6	37	2.9	A
R56	2157.7	1.85	1.51	228	20	4.6	A
R57	1765.8	2.01	2.17	94	47	4.4	A
R58	1875.7	2.01	1.89	356	30	10.7	A
R59	2451.3	2.05	1.98	248	30	7.4	A
R60	2746.1	2.04	2.04	326	30	9.8	A
R61	3689.1	2.12	2.02	107.3	37	4	A
R62	2265.7	2.15	2.26	120	45	9	A
R63	247.4	1.97	2.24	218	38	8.3	A
R64	4151.9	2.14	2.22	242	45	10.9	A
R65	1825.7	2.12	2.24	147	45	6.6	A
R66	1654.1	2.13	2.19	88	45	4	A
R67	2447.1	1.99	1.82	183	31	5.7	A
R68	2139.2	2.16	2.3	210	45	9.5	A
R69	3485.2	2.14	2.13	242	45	10.9	A
R70	3324	2.21	2.35	234	45	10.5	A
R71	2300.6	2.06	2.2	134	47	6.3	A
R72	1578.3	2.14	2.28	202	28	6.6	A
R73	177.2	1.8	1.07	143	20	2.9	A
R74	1977.8	2	1.92	88.4	47	3.8	A
R75	1697.7	2.12	2.07	171	47	8	A
R76	2202.9	2.09	1.9	158.2	37	5.9	A
R77	1720.8	2.09	2.03	140	37	5.2	A
R78	3215.1	2.12	1.99	96.88	60	5.8	A
R79	2039	2.16	2.27	312	45	14	A
R80	1183.7	2.03	1.99	193	32	6.2	A
R81	1905.1	2	2.27	202	47	9.5	A
R82	2514.4	2.09	2.19	123	47	5.8	A
R83	1154.5	2.14	2.05	266	45	5.3	A
R84	2279.2	1.85	1.56	372	47	5.3	A
R85	3324	2.09	2.12	312	45	14	A
R86	2622.4	1.95	1.98	256	47	12	A
R87	2883.1	2.13	2.14	234	45	10.5	A
R88	2476.8	1.77	1.88	228	20	4.6	A
R89	1303.2	2.06	2.12	110	47	5.2	A
R90	2736	1.97	2.06	252	47	11.8	A

[1]When a sample could not complete the standard sequencing requirement, those samples were discarded, and genomic DNA was extracted from the same colony samples.
