# Supplementary material for: Discovery of SNP Molecular Markers and Candidate Genes Associated with Sacbrood Virus Resistance in Apis cerana cerana Larvae by Whole-Genome Resequencing
Source: Int J Mol Sci. 2023 Mar 25;24(7):6238. doi: 10.3390/ijms24076238 (PMC10094193; doi:10.3390/ijms24076238)
Supplement: Supplementary file 1 [file ijms-24-06238-s001.zip › Table S20 Primer pairs used in PCRs to validate SNP and RT-PCR for virus detection and quantification..pdf]

Table S20 Primer pairs used in PCRs to validate SNP and RT-PCR for virus detection and quantification.

Scaffold ID and SNP Position	Primer type	Orientation	Sequences	Start	Length	Tm	Product Sizes	References
KZ288481.1_329501	SNP flanking	Forward	TGGCTCCATAGCATCTTTCA	437	20	59.38	285	In this study
KZ288481.1_329501	SNP flanking	Reverse	TCAAATTTTGGATTTGACCTGA	721	22	59.43		
KZ288481.1_344840	SNP flanking	Forward	GTGCTCCCTTCTTGGTGAGT	309	20	59.3	253	In this study
KZ288481.1_344840	SNP flanking	Reverse	AACCTCAAAAATGCGATAAGAGA	561	23	59.3		
KZ288481.1_359439	SNP flanking	Forward	TTGCGTCAAATCTGAAAAATCA	412	22	60.6	237	In this study
KZ288481.1_359439	SNP flanking	Reverse	CACCCAAATAAACTTGTTATCTTTTT	648	26	58.23		
KZ288481.1_397572	SNP flanking	Forward	TGGCTAAATAAATTGTATTGTGTGAA	382	26	58.99	190	In this study
KZ288481.1_397572	SNP flanking	Reverse	TTTTGCAATAAATGCTAGTTTCAATC	571	26	59.94		
KZ288481.1_417529	SNP flanking	Forward	TTTCTAAAGCACTATTACCATCTTTTT	291	27	57.52	237	In this study
KZ288481.1_417529	SNP flanking	Reverse	TTCTTAACATCACAACAAGTTGCAG	527	25	60.59		
KZ288481.1_422152	SNP flanking	Forward	CTCCGGAAATGATCCAGATT	287	20	58.93	250	In this study
KZ288481.1_422152	SNP flanking	Reverse	CATCTTGTCAAAACAGTTCCGTTA	536	24	60.43		
KZ288481.1_423535	SNP flanking	Forward	TGTCAATGAAGAAAATCGCTGT	476	22	59.75	121	In this study
KZ288481.1_423535	SNP flanking	Reverse	TTTTCAGCCAACAACACTCG	596	20	59.88		
KZ288495.1_22286	SNP flanking	Forward	TTTTATGTATGTATTGGATGAAAAATG	352	27	57.65	259	In this study
KZ288495.1_22286	SNP flanking	Reverse	GCACAATAACCGTGATGGAC	610	20	58.85		
KZ288481.1_322476	SNP flanking	Forward	TGGAACTACCAGGAAGAGGGTA	271	22	59.99	717	In this study
KZ288481.1_322476	SNP flanking	Reverse	TCCTGTTGCCATCTTTAACAAA	987	22	59.63		
KZ288474.1_322717	SNP flanking	Forward	TTATTATTCGCAGCGTGTCG	51	20	59.86	679	In this study
KZ288474.1_322717	SNP flanking	Reverse	GCTTGCAGCACATCCACTAA	729	20	60.02		
KZ288479.1_95621	SNP flanking	Forward	CCTTGTTCCTTTCTACCGTCA	85	21	59.22	654	In this study
KZ288479.1_95621	SNP flanking	Reverse	AAACATGCATTTGCACGAAG	738	20	59.74		
KZ288474.1_222009	SNP flanking	Forward	TTGATCAATCAAACAATAGCAAA	277	23	57.42	674	In this study
KZ288474.1_222009	SNP flanking	Reverse	ATTCCCACTAATGGGCTGGT	950	20	60.58		
KZ288285.1_94887	SNP flanking	Forward	AATGCCACAATGAACATCCA	246	20	59.78	699	In this study
KZ288285.1_94887	SNP flanking	Reverse	AAGGCAGATACATGCGATCA	944	20	59.26		
KZ288285.1_96634	SNP flanking	Forward	CTGGTACCGCTGGTCAGACT	213	20	60.32	696	In this study

KZ288285.1_96634	SNP flanking	Reverse	GATTGCATCTGAGGCTGTGA	908	20	59.95		
KZ288285.1_3383	SNP flanking	Forward	ATAGCATACGTAATGAAGTC	617	20	57.23	402	In this study
KZ288285.1_3383	SNP flanking	Reverse	AATTTGGCAAGTTACTACAA	215	20	58.22		
KZ288285.1_96902	SNP flanking	Forward	GTTGGTCATCCAGCTCCTG	1	19	59.2	697	In this study
KZ288285.1_96902	SNP flanking	Reverse	TTGAACAAGACCTGCTGCTG	697	20	60.18		
SBV1	Virus detection	Forward	ACCAACCGATTCCTCAGTAG	221	20	57	487	
SBV1	Virus detection	Reverse	CCTTGGAACTCTGCTGTGTA	708	20	57		
SBV2	Virus detection	Forward	AATGGTGCGGTGGACTATGG	8038	20	57	597	
SBV2	Virus detection	Reverse	TGATACAGAGCGGCTCGACA	8635	20	57		
SBV374F	Quantification	Forward	CAG TGG ACT CTT ATA CCG ATT TG		23	58		
SBV551Rd:	“	Reverse	GAG GTA ATA ACT TTT CGC CAY ACT A		25	58	178	
SBV469F probe	“	Forward	FAM-GAC GAA GAA TCT GGA ATG T-MGB)					
Api cerana β- actin	Internal control	Forward	AATTTTCATGGTGGATGGTGC		21	60	181	
Api cerana β- actin	Internal control	Reverse	AGGAATGGAAGCTTGCGGTA		20	60		
