# Supplementary material for: Discovery of SNP Molecular Markers and Candidate Genes Associated with Sacbrood Virus Resistance in Apis cerana cerana Larvae by Whole-Genome Resequencing
Source: Int J Mol Sci. 2023 Mar 25;24(7):6238. doi: 10.3390/ijms24076238 (PMC10094193; doi:10.3390/ijms24076238)
Supplement: Supplementary file 1 [file ijms-24-06238-s001.zip › Table S3 Sample sequences information and data filter statistics results..pdf]

Table S3 Sample sequences information and data filter statistics results.

ID	Sample name	Raw reads	Adapter-related in (%)	Inferior in (%)	Clean reads
R01	S.1-1	16,701,794	0.06	0.00	16,691,189
R02	S.1-2	10,383,104	0.10	0.00	10,372,441
R03	S.1-3	14,841,181	0.07	0.00	14,829,892
R04	S.1-9	12,752,528	0.08	0.00	12,741,886
R05	S.1-10	14,577,539	0.10	0.00	14,562,881
R06	R. 1-1	11,683,434	0.07	0.00	11,674,822
R07	R.1-2	10,470,953	0.09	0.00	10,461,561
R08	R.1-3	13,350,188	0.07	0.00	13,339,896
R09	R.1-4	13,213,389	0.12	0.00	13,197,212
R10	R.1-6	13,136,448	0.06	0.00	13,128,662
R11	R.1-7	13,005,471	0.07	0.00	12,995,456
R12	R.1-9	12,496,317	0.09	0.00	12,484,431
R13	S.2-2	13,491,913	0.07	0.00	13,481,480
R14	S.2-3	13,499,676	0.05	0.00	13,492,426
R15	S.2-4	14,892,890	0.06	0.00	14,882,977
R16	S.2-5	12,140,657	0.07	0.00	12,132,045
R17	S.2-6	12,574,909	0.06	0.01	12,566,850
R18	S.2-7	13,742,525	0.09	0.00	13,730,177
R19	S.2-10	12,145,136	0.05	0.01	12,137,940
R20	S.2-13	12,197,271	0.14	0.01	12,179,413
R21	S.2-14	12,241,754	0.07	0.01	12,232,328
R22	R.2-1	12,750,484	0.06	0.00	12,742,842
R23	R.2-2	12,256,279	0.05	0.01	12,249,786
R24	R.2-3	12,303,242	0.05	0.01	12,296,077
R25	R.2-4	11,530,944	0.04	0.01	11,525,077
R26	R.2-5	12,135,115	0.03	0.01	12,130,178
R27	R.2-7	12,780,471	0.07	0.01	12,770,346
R28	R.2-11	11,412,291	0.07	0.01	11,403,938
R29	R.2-13	13,918,387	0.10	0.00	13,904,616
R30	R.2-14	12,066,331	0.09	0.01	12,055,712
R31	R.2-15	12,881,130	0.11	0.01	12,866,047
R32	S.3-1	9,671,350	0.11	0.00	9,660,878
R33	S.3-3	11,956,138	0.03	0.01	11,951,380
R34	S.3-4	13,669,461	0.09	0.00	13,656,972
R35	S.3-5	10,680,105	0.07	0.01	10,671,409
R36	S.3-6	12,680,767	0.05	0.01	12,673,278
R37	S.3-7	14,692,830	0.04	0.01	14,686,710
R38	S.3-10	11,946,334	0.05	0.01	11,939,169
R39	S.3-12	13,073,316	0.04	0.01	13,067,226
R40	S.3-13	11,470,492	0.04	0.01	11,465,098
R41	R.3-1	11,449,920	0.04	0.01	11,444,584
R42	R.3-2	11,717,471	0.04	0.01	11,712,619
R43	R.3-4	11,246,180	0.08	0.01	11,235,953
R44	R.3-5	10,343,056	0.04	0.01	10,338,503
R45	R.3-8	11,665,803	0.04	0.01	11,660,285
R46	R.3-9	10,553,624	0.04	0.01	10,548,969
R47	R.3-10	13,217,793	0.04	0.01	13,212,301
R48	R.3-13	13,204,220	0.04	0.01	13,198,583
R49	S.1-4	11,027,431	0.07	0.01	11,018,656

R50	S.1-5	11,084,015	0.06	0.01	11,077,036
R51	S.1-8	13,219,461	0.06	0.00	13,210,917
R52	R.1-5	11,972,388	0.05	0.01	11,965,626
R53	R.1-8	11,202,177	0.07	0.01	11,193,385
R54	R.1-10	12,385,817	0.06	0.01	12,377,979
R55	R.1-11	12,223,184	0.06	0.01	12,215,574
R56	R.1-12	12,813,307	0.05	0.01	12,805,702
R57	R.1-13	11,844,917	0.06	0.01	11,837,031
R58	R.1-14	11,061,351	0.05	0.01	11,055,444
R59	R.1-15	11,885,650	0.06	0.01	11,877,841
R60	S.2-9	10,615,170	0.03	0.01	10,611,190
R61	S.2-11	10,953,513	0.05	0.01	10,947,749
R62	S.2-15	12,501,081	0.06	0.01	12,493,159
R63	R.2-6	13,925,582	0.05	0.00	13,918,094
R64	R.2-8	12,718,593	0.07	0.00	12,709,017
R65	R.2-9	13,256,109	0.05	0.00	13,249,449
R66	R.2-12	13,171,850	0.06	0.00	13,163,162
R67	S.3-11	13,410,665	0.05	0.00	13,403,432
R68	S.3-14	13,973,457	0.07	0.00	13,963,466
R69	S.3-15	14,649,204	0.04	0.00	14,642,258
R70	R.3-3	15,302,709	0.06	0.00	15,293,208
R71	R.3-6	14,981,175	0.06	0.00	14,972,276
R72	R.3-12	12,660,694	0.06	0.00	12,653,054
R73	R.3-7	11,293,034	0.16	0.01	11,274,540
R74	R.3-11	11,424,476	0.07	0.01	11,415,149
R75	R.3-14	11,251,034	0.07	0.01	11,242,320
R76	R.3-15	13,083,335	0.07	0.00	13,073,668
R77	S.1-7	13,203,241	0.08	0.00	13,192,476
R78	S.1-6	13,754,734	0.07	0.00	13,745,224
R79	S.1-11	13,640,237	0.08	0.00	13,629,754
R80	S.1-12	11,491,770	0.10	0.00	11,480,285
R81	S.1-13	12,975,604	0.15	0.00	12,956,731
R82	S.1-14	12,867,338	0.14	0.00	12,849,451
R83	S.1-15	14,174,095	0.10	0.00	14,160,619
R84	S.2-1	12,073,169	0.06	0.01	12,064,398
R85	S.2-8	11,558,395	0.11	0.00	11,546,199
R86	S.2-12	13,446,574	0.12	0.00	13,430,940
R87	R.2-10	11,036,784	0.08	0.01	11,026,795
R88	S.3-2	11,365,986	0.07	0.01	11,357,871
R89	S.3-8	12,836,739	0.17	0.00	12,814,679
R90	S.3-9	12,645,460	0.18	0.01	12,622,283

[1]Sample ID: Sample number; a unified number of project samples; raw reads: initial sequencing reads; adapter related: number of reads with connectors filtered; inferior percentage: N content of more than 10% of reads and quality values are lower than. The base of 10 exceeds 50% of the reads ratio; clean reads: the number of reads remaining after filtering.
