# Supplementary material for: Discovery of SNP Molecular Markers and Candidate Genes Associated with Sacbrood Virus Resistance in Apis cerana cerana Larvae by Whole-Genome Resequencing
Source: Int J Mol Sci. 2023 Mar 25;24(7):6238. doi: 10.3390/ijms24076238 (PMC10094193; doi:10.3390/ijms24076238)
Supplement: Supplementary file 1 [file ijms-24-06238-s001.zip › Table S4 Predicting the probability of error in base discrimination..pdf]

Table S4 Predicting the probability of error in base discrimination.

Phred-score	Incorrect base recognition	The correct recognition rate of the base group	Q-score
10	1/10	90%	Q10
20	1/100	99%	Q20
30	1/1000	99.90%	Q30
40	1/10000	99.99%	Q40
