# Supplementary material for: Discovery of SNP Molecular Markers and Candidate Genes Associated with Sacbrood Virus Resistance in Apis cerana cerana Larvae by Whole-Genome Resequencing
Source: Int J Mol Sci. 2023 Mar 25;24(7):6238. doi: 10.3390/ijms24076238 (PMC10094193; doi:10.3390/ijms24076238)
Supplement: Supplementary file 1 [file ijms-24-06238-s001.zip › Table S5 Sample sequences data assessment and quality control..pdf]

Table S5 Sample sequences data assessment and quality control.

ID	Raw _Reads	Clean Reads	Clean Base	Q20 (%)	Q30 (%)	GC (%)
R01	16701794	16691189	4997764254	97.3	92.53	34.33
R02	10383104	10372441	3102700210	97.09	92.18	34.14
R03	14841181	14829892	4438886352	96.93	91.78	34.48
R04	12752528	12741886	3812880768	97.18	92.32	34.48
R05	14577539	14562881	4358898238	97.2	92.31	33.72
R06	11683434	11674822	3496511262	96.77	91.42	32.93
R07	10470953	10461561	3132323894	97.6	93.2	33.72
R08	13350188	13339896	3995537088	96.92	91.74	33.56
R09	13213389	13197212	3953210844	97.08	92.15	34.12
R10	13136448	13128662	3932175704	97.33	92.65	34.33
R11	13005471	12995456	3891202770	97.32	92.63	35.21
R12	12496317	12484431	3738751218	97.19	92.37	34.49
R13	13491913	13481488	4036647382	97.16	92.33	36.23
R14	13499676	13492426	4041387252	97.01	91.98	36.6
R15	14892890	14882977	4456820948	97.41	92.85	37.06
R16	12140657	12132045	3634497296	95.56	89.04	34.42
R17	12574909	12566850	3762624060	97.72	93.47	35.12
R18	13742525	13730177	4111935372	97.03	92.02	34.5
R19	12145136	12137940	3636179214	97.42	92.8	34.77
R20	12197271	12179413	3640497460	97.54	93.12	35.73
R21	12241754	12232328	3661893398	97.71	93.42	34.4
R22	12750484	12742842	3816688812	97.2	92.34	34.12
R23	12256279	12249786	3669130664	97.47	92.9	33.53
R24	12303242	12296077	3682829672	97.56	93.12	34.32
R25	11530944	11525077	3452070422	97.55	93.13	34.51
R26	12135115	12130178	3633907776	97.5	92.98	34.1
R27	12788471	12770346	3824778646	97.31	92.7	34.67
R28	11412291	11403938	3414657408	97.35	92.64	33.64
R29	13918387	13904616	4165578530	96.65	91.19	34.36
R30	12066331	12055712	3610859004	97.22	92.45	34.25
R31	12881130	12866047	3849832488	97.43	93.08	35.97
R32	9671350	9660878	2891289120	97.47	93.03	31.87
R33	11956138	11951388	3588335188	96.86	91.31	34.29
R34	13669461	13656972	4090778810	97.74	93.59	35.08
R35	10688105	10671409	3190642382	97.68	93.45	34.7
R36	12688767	12673278	3795179066	97.51	93.01	34.56
R37	14692830	14686710	4399616312	97.37	92.63	34.13
R38	11946334	11939169	3574003338	97.47	92.93	32.3
R39	13073316	13067226	3914623748	97.71	93.41	34.88
R40	11470492	11465098	3433662478	97.58	93.17	34.45
R41	11449920	11444584	3428688454	97.55	93.1	34.09
R42	11717471	11712619	3508375732	97.63	93.29	34.55
R43	11246188	11235953	3363358558	97.34	92.66	31.9
R44	10343056	10338503	3096848484	97.65	93.27	33.44
R45	11665883	11660285	3493154524	97.44	92.83	34.38
R46	10553624	10548969	3159900232	97.66	93.33	34.28
R47	13217793	13212301	3957927390	97.51	93.03	34.94
R48	13204220	13198583	3953257704	97.64	93.27	34.13
R49	11027431	11018656	3297828568	97.24	92.46	34.09
R50	11084015	11077036	3317558852	97.34	92.69	34.6
R51	13219461	13210917	3956402290	97.25	92.46	34.4

R52	11972388	11965626	3582797462	97.62	93.28	34.28
R53	11202177	11193385	3352096460	97.25	92.51	34.76
R54	12385817	12377979	3706065362	97.5	92.94	33.11
R55	12223184	12215574	3656539014	97.47	92.86	34.24
R56	12813307	12885702	3834507298	97.53	93.03	34.22
R57	11844917	11837031	3544388644	97.55	93.09	33.9
R58	11061351	11055444	3310703164	97.61	93.18	33.97
R59	11885650	11877841	3556629520	97.3	92.52	33.61
R60	10615170	10611190	3179107928	97.47	92.8	32.75
R61	10953513	10947749	3279621990	97.34	92.63	34.24
R62	12501081	12493159	3740533896	97.68	93.37	33.98
R63	13925582	13918894	4168486240	97.25	92.42	34.04
R64	12718593	12709017	3886702664	96.55	91.04	34.03
R65	13256109	13249449	3968396676	97.33	92.59	34.2
R66	13171850	13163162	3943442990	97.11	92.19	34.79
R67	13410665	13403432	4015717900	97.37	92.62	33.37
R68	13973457	13963466	4181029298	97.26	92.49	34.27
R69	14649204	14642258	4386175056	97.22	92.36	33.81
R70	15302709	15293208	4581636682	97.1	92.1	34.08
R71	14981175	14972276	4485371894	96.99	91.89	34.19
R72	12660694	12653054	3790018652	97.13	92.16	32.87
R73	11293034	11274540	3370639278	97.2	92.37	31.48
R74	11424476	11415149	3415841662	97.44	92.89	32.31
R75	11251034	11242320	3364526172	97.14	92.24	33.17
R76	13083335	13073668	3915729400	97.28	92.44	33.3
R77	13203241	13192476	3952255604	97.19	92.28	33.65
R78	13754734	13745224	4118120882	97.1	92.08	33.84
R79	13640237	13629754	4082692474	97.94	94.05	34.22
R88	11491770	11488285	3438678800	97.89	93.88	33.07
R81	12975604	12956731	3877931062	97.71	93.5	33.4
R82	12867338	12849451	3848485296	97.77	93.69	34.08
R83	14174095	14160619	4241023678	97.91	94	34.4
R84	12073169	12064398	3612090366	97.22	92.44	34.67
R85	11558395	11546199	3458404630	98	94.2	33.72
R86	13446574	13430940	4023321386	97.74	93.61	33.81
R87	11036784	11026795	3303637682	97.61	93.15	34.02
R88	11365986	11357871	3402995494	97.48	92.9	35.32
R89	12836739	12814679	3838359820	97.95	94.16	34.97
R90	12645460	12622283	3779459842	97.83	93.65	35.2

[1]ID: uniform number of project samples for project; raw reads: number of reads for initial sequence, number of pair-end sequences in four behavior units; clean reads: number of reads after filtration, calculated by raw reads; clean bases: the number of bases after filtration, number of clean reads multiplied by sequence length; Q20 (%): number of bases with a quality value greater than or equal to 20 is the number of Q30 (%): the percentage of bases whose mass value is greater than or equal to 30 to the total number of bases; GC (%): GC content of samples, that is, the percentage of G and C type bases to the total number of bases.
