# Supplementary material for: Discovery of SNP Molecular Markers and Candidate Genes Associated with Sacbrood Virus Resistance in Apis cerana cerana Larvae by Whole-Genome Resequencing
Source: Int J Mol Sci. 2023 Mar 25;24(7):6238. doi: 10.3390/ijms24076238 (PMC10094193; doi:10.3390/ijms24076238)
Supplement: Supplementary file 1 [file ijms-24-06238-s001.zip › Table S6 Sample coverage depth and coverage ratio statistics..pdf]

Table S6 Samples coverage depth and coverage ratio statistics.

ID	Ave_depth	Cov_ratio_1X (%)	Cov_ratio_5X (%)	Cov_ratio_10X (%)
R01	20	99.33	96.94	90.98
R02	12	99.26	92.63	68.07
R03	17	99.49	96.79	87.58
R04	15	99.2	95.05	80.78
R05	17	99.5	96.72	86.47
R06	13	99.52	94.9	73.63
R07	12	99.36	93.4	68.09
R08	16	99.56	96.6	84.35
R09	15	99.18	95.26	81.39
R10	15	98.58	93.87	79.37
R11	16	97.82	92.54	79.75
R12	15	98.87	94.1	78.79
R13	17	96.66	91.09	80.73
R14	17	94.65	88.89	79.31
R15	16	98.48	94.23	82.49
R16	14	99.25	94.99	77.98
R17	14	99.12	94.43	76.37
R18	16	99.43	96.26	84.49
R19	15	98.98	94.44	78.94
R20	15	98.14	91.99	75.87
R21	15	98.61	93.74	78.49
R22	15	99.05	94.95	80.23
R23	14	99.08	94.52	77.38
R24	14	99.11	94.34	76.93
R25	13	98.88	93.42	74.11
R26	14	99.18	94.97	78.74
R27	15	98.85	94.29	79.2
R28	13	99.23	94.05	73.36
R29	16	99.46	96.31	84.89
R30	14	98.97	93.81	76.11
R31	15	98.15	91.79	75.85
R32	11	99.44	91.4	62.49
R33	14	99.15	94.67	77.51
R34	17	98.62	94.39	82.57
R35	12	98.42	90.81	67.8
R36	15	99.01	94.36	78.85
R37	18	99.49	96.98	88.13
R38	14	99.18	94.25	75.57
R39	16	98.93	94.95	82.21
R40	12	98.64	91.35	67.2
R41	13	99	93.67	73.41
R42	14	98.63	93.02	74.54
R43	13	99.51	93.71	69.99
R44	12	99.09	92.25	65.65
R45	13	99.01	93.63	74.02
R46	12	98.92	92.19	67.99
R47	16	98.7	94.18	80.89
R48	15	99.02	94.9	80.98
R49	13	99.23	93.59	72.7
R50	13	99.25	93.59	72.29

R51	15	99.19	95.15	80.86
R52	14	99.03	93.96	75.87
R53	13	98.73	92.25	71.28
R54	14	99.31	94.73	76.62
R55	14	99.2	94.51	76.66
R56	15	99.25	94.94	78.82
R57	13	99.16	93.8	74.06
R58	13	99.44	93.99	72.26
R59	14	99.32	94.64	75.92
R60	12	99.42	93.96	69.68
R61	13	99.32	93.73	70.61
R62	14	99.38	95.19	78.89
R63	16	99.56	96.72	85.37
R64	15	99.48	95.7	80.17
R65	16	99.34	95.86	83.64
R66	16	99.1	95.16	82.23
R67	16	99.55	96.93	85.94
R68	16	99.41	96.26	85
R69	18	99.52	97.14	89.12
R70	18	99.55	97.17	89.41
R71	18	99.3	96.42	87.69
R72	14	99.36	95.43	78.49
R73	13	99.54	93.65	70.61
R74	13	99.34	93.26	70.62
R75	13	99.16	93.15	71.21
R76	15	99.51	96.47	83.5
R77	15	99.43	96.23	83.4
R78	16	99.41	96.36	85.47
R79	16	98.95	95.07	82.52
R80	13	99.5	94.82	74.36
R81	15	99.2	94.87	78.98
R82	15	99.04	94.66	79.69
R83	17	98.78	95.15	84.86
R84	14	98.86	93.44	74.86
R85	14	99.12	94.26	75.64
R86	16	99.33	95.92	82.92
R87	13	98.88	93.05	71.01
R88	13	99.07	93.69	72.5
R89	15	98.72	94.19	80.49
R90	15	98.7	93.91	78.6

[1]ID: The unified number of the project sample; Ave-depth: the average coverage depth of the sample; Coverage: the proportion of base numbers with a given coverage depth or more to the total base number of the reference genome. The mapping was carried out according to the coverage depth of each chromosome point. If the coverage depth was evenly distributed on the chromosome, the randomness of sequencing could be considered as good.
