# Supplementary material for: Discovery of SNP Molecular Markers and Candidate Genes Associated with Sacbrood Virus Resistance in Apis cerana cerana Larvae by Whole-Genome Resequencing
Source: Int J Mol Sci. 2023 Mar 25;24(7):6238. doi: 10.3390/ijms24076238 (PMC10094193; doi:10.3390/ijms24076238)
Supplement: Supplementary file 1 [file ijms-24-06238-s001.zip › Table S7 Mapping ratio based on the comparison to the genome of 90 accessions Apis cerana cerana..pdf]

**Table S7** Mapping ratio based on the comparison to the reference genome of 90 accessions *Apis cerana cerana.*

| ID | Group | Total _Reads | Mapped (%) | Properly Mapped (%) |
|---|---|---|---|---|
| R01 | S_1_ 1 | 33382378 | 89.56 | 85.13 |
| R02 | S_1_ 2 | 20744882 | 89.38 | 79.44 |
| R03 | S_1_ 3 | 29659784 | 87.68 | 82.09 |
| R49 | S_ 1_ 4 | 22037312 | 90.00 | 88.71 |
| R50 | S_1_ 5 | 22154072 | 88.83 | 79.69 |
| R78 | S_1_ 6 | 27490448 | 88.00 | 88.45 |
| R77 | S_1_ 7 | 26384952 | 87.27 | 79.90 |
| R51 | S_1_ 8 | 26421834 | 87.07 | 81.83 |
| R04 | S_1_ 9 | 25483772 | 89.88 | 83.98 |
| R05 | S_1_10 | 29125762 | 87.46 | 82.04 |
| R79 | S_1_11 | 27259508 | 89.41 | 84.63 |
| R88 | S_1_12 | 22960570 | 88.84 | 81.38 |
| R81 | S_1_13 | 25913462 | 87.55 | 81.31 |
| R82 | S_1_14 | 25698902 | 88.57 | 81.34 |
| R83 | S_1_15 | 28321238 | 90.09 | 84.64 |
| R06 | R_ 1_1 | 23349644 | 86.08 | 75.99 |
| R07 | R_1_ 2 | 20923122 | 87.49 | 79.25 |
| R08 | R_1_ 3 | 26679792 | 88.56 | 81.27 |
| R09 | R_1_ 4 | 26394424 | 86.65 | 79.43 |
| R52 | R_ 1_ 5 | 23931252 | 87.92 | 81.62 |
| R10 | R_1_ 6 | 26257324 | 86.62 | 88.96 |
| R11 | R_ 1_ 7 | 25990912 | 88.69 | 83.47 |
| R53 | R_ 1_ 8 | 22386770 | 87.97 | 88.74 |
| R12 | R_ 1_ 9 | 24968862 | 88.29 | 88.82 |
| R54 | R_ 1_10 | 24755958 | 86.20 | 77.29 |
| R55 | R_1_11 | 24431148 | 86.94 | 81.04 |
| R56 | R_ 1_12 | 25611404 | 86.71 | 88.83 |
| R57 | R_ 1_13 | 23674062 | 87.00 | 79.68 |
| R58 | R_ 1_14 | 22110888 | 89.58 | 82.43 |
| R59 | R_ 1_15 | 23755682 | 87.88 | 79.70 |
| R84 | S_ 2_1 | 24128796 | 86.30 | 81.49 |
| R13 | S_ 2_ 2 | 26962960 | 90.73 | 85.89 |
| R14 | S_ 2_ 3 | 26984852 | 89.00 | 85.47 |
| R15 | S_ 2_ 4 | 29765954 | 81.13 | 77.72 |
| R16 | S_ 2_ 5 | 24264090 | 87.37 | 81.48 |
| R17 | S_ 2_ 6 | 25133700 | 83.70 | 88.30 |
| R18 | S_ 2_ 7 | 27460354 | 87.82 | 82.10 |
| R85 | S_ 2_ 8 | 23092398 | 89.46 | 82.66 |
| R60 | S_ 2_ 9 | 21222388 | 88.40 | 81.46 |
| R19 | S_ 2_10 | 24275888 | 90.18 | 85.78 |
| R61 | S_ 2_11 | 21895498 | 86.56 | 77.51 |
| R86 | S_ 2_12 | 26861888 | 87.99 | 88.35 |
| R20 | S_ 2_13 | 24358826 | 89.89 | 85.54 |
| R21 | S_ 2_14 | 24464656 | 89.51 | 85.28 |
| R62 | S_ 2_ 15 | 24986318 | 87.69 | 79.20 |
| R22 | R_ 2_1 | 25485684 | 87.62 | 82.65 |
| R23 | R_ 2_ 2 | 24499572 | 87.46 | 81.23 |
| R24 | R_ 2_ 3 | 24592154 | 87.27 | 81.22 |
| R25 | R_ 2_ 4 | 23050154 | 88.19 | 82.51 |
| R26 | R_ 2_ 5 | 24260356 | 89.31 | 83.65 |

| | | | | |
|---|---|---|---|---|
| R63 | R_2_6 | 27836188 | 88.23 | 82.68 |
| R27 | R_2_7 | 25540692 | 87.94 | 88.91 |
| R64 | R_2_8 | 25418834 | 87.08 | 78.54 |
| R65 | R_2_9 | 26498898 | 90.14 | 84.87 |
| R87 | R_2_10 | 22053590 | 87.03 | 88.53 |
| R28 | R_2_11 | 22887876 | 87.89 | 88.89 |
| R66 | R_2_12 | 26326324 | 88.09 | 81.71 |
| R29 | R_2_13 | 27889232 | 87.46 | 79.37 |
| R30 | R_3_14 | 24111424 | 87.51 | 79.27 |
| R31 | R_2_15 | 25732094 | 86.61 | 81.42 |
| R32 | S_3_1 | 19321756 | 89.89 | 88.43 |
| R88 | S_3_2 | 22715742 | 84.88 | 78.98 |
| R33 | S_3_3 | 23902760 | 88.70 | 83.00 |
| R34 | S_3_4 | 27313944 | 90.27 | 85.4 |
| R35 | S_3_5 | 21342818 | 88.53 | 83.82 |
| R36 | S_3_6 | 25346556 | 87.99 | 81.27 |
| R37 | S_3_7 | 29373420 | 89.36 | 84.16 |
| R89 | S_3_8 | 25629358 | 89.64 | 84.63 |
| R90 | S_3_9 | 25244566 | 86.41 | 82.34 |
| R38 | S_3_10 | 23878338 | 89.49 | 85.25 |
| R67 | S_3_11 | 26886864 | 89.73 | 83.08 |
| R39 | S_3_12 | 26134452 | 89.67 | 84.87 |
| R40 | S_3_13 | 22930196 | 81.03 | 75.11 |
| R68 | S_3_14 | 27926932 | 87.79 | 81.15 |
| R69 | S_3_15 | 29284516 | 90.51 | 84.54 |
| R41 | R_3_1 | 22889168 | 87.32 | 81.17 |
| R42 | R_3_2 | 23425238 | 88.25 | 83.02 |
| R70 | R_3_3 | 30586416 | 88.34 | 82.08 |
| R43 | R_3_4 | 22471906 | 86.68 | 78.88 |
| R44 | R_3_5 | 20677006 | 86.99 | 88.62 |
| R71 | R_3_6 | 29944552 | 87.87 | 82.41 |
| R73 | R_3_7 | 22549088 | 87.81 | 75.49 |
| R45 | R_3_8 | 23320570 | 86.91 | 88.28 |
| R46 | R_3_9 | 21097938 | 88.30 | 83.03 |
| R47 | R_3_10 | 26424602 | 88.19 | 83.25 |
| R74 | R_3_11 | 22830298 | 87.27 | 82.39 |
| R72 | R_3_12 | 25306108 | 85.81 | 79.17 |
| R48 | R_3_13 | 26397166 | 87.27 | 82.07 |
| R75 | R_3_14 | 22484640 | 88.23 | 82.37 |
| R76 | R_3_15 | 26147336 | 88.20 | 82.11 |
