# Supplementary material for: Discovery of SNP Molecular Markers and Candidate Genes Associated with Sacbrood Virus Resistance in Apis cerana cerana Larvae by Whole-Genome Resequencing
Source: Int J Mol Sci. 2023 Mar 25;24(7):6238. doi: 10.3390/ijms24076238 (PMC10094193; doi:10.3390/ijms24076238)
Supplement: Supplementary file 1 [file ijms-24-06238-s001.zip › Table S8 Whole-genome and coding regions of insertion and deletion (InDel) statistics..pdf]

Table S8 Whole-genome and coding regions of insertions and deletions (InDels) statistics.

Sample ID	CDS Insertion	CDS Deletion	CDS_ Homo	CDS_ Het	CDS Total	Genome_ insertion	Genome_ Deletion	Genome_ Homo	Genome_ Het	Genome_ Total
R01	372	393	264	501	765	61513	68395	40180	89728	129908
R02	326	340	273	393	666	56496	62554	43808	75242	119050
R03	357	358	254	461	715	60175	67136	41107	86204	127311
R04	352	385	270	467	737	59171	66181	41594	83758	125352
R05	353	352	277	428	705	60202	66925	41417	85710	127127
R06	347	370	268	449	717	57660	64525	43034	79151	122185
R07	343	353	276	420	696	57977	64503	42942	79538	122480
R08	337	366	279	424	703	59529	66383	41506	84406	125912
R09	354	339	262	431	693	59375	65809	41705	83479	125184
R10	359	350	266	443	709	58294	65026	41776	81544	123320
R11	333	349	270	412	682	58482	65001	41317	82166	123483
R12	333	363	290	406	696	58251	64511	42146	80616	122762
R13	355	357	245	467	712	58088	64600	39741	82947	122688
R14	349	359	258	450	708	57108	63324	39497	80935	120432
R15	335	351	255	431	686	58981	65204	41273	82912	124185
R16	341	339	268	412	680	57848	64181	42312	79717	122029
R17	352	365	257	460	717	58321	64737	42529	80529	123058
R18	347	382	263	466	729	60139	66902	40861	86180	127041
R19	334	345	267	412	679	58307	64997	41572	81732	123304
R20	334	346	273	407	680	57578	64094	42002	79670	121672
R21	348	353	272	429	701	58618	65097	41731	81984	123715
R22	354	352	254	452	706	58890	65396	42144	82142	124286
R23	319	352	263	408	671	58534	64882	42292	81124	123416
R24	325	349	245	429	674	58992	65454	42314	82132	124446
R25	328	336	265	399	664	57951	64526	42534	79943	122477
R26	351	357	277	431	708	58714	65270	42114	81870	123984
R27	343	361	270	434	704	58641	64921	41964	81598	123562
R28	326	348	265	409	674	57410	63375	43015	77770	120785
R29	332	375	252	455	707	60378	66627	41184	85821	127005
R30	344	368	253	459	712	58317	64944	42064	81197	123261
R31	325	364	278	411	689	57473	63849	41909	79413	121322
R32	332	351	261	422	683	55490	61625	44335	72780	117115
R33	341	363	261	443	704	58809	65389	42401	81797	124198
R34	333	352	264	421	685	58902	65671	43109	81464	124573
R35	332	372	286	418	704	57078	63780	42664	78194	120858
R36	337	386	260	463	723	59189	65817	41521	83485	125006
R37	360	384	251	493	744	60776	67703	40993	87486	128479
R38	277	262	472	67	539	45795	49182	83048	11929	94977
R39	315	343	258	400	658	59533	65879	43043	82369	125412
R40	273	244	463	54	517	44968	48679	84052	9595	93647
R41	344	361	255	450	705	58104	64862	42685	80281	122966
R42	339	374	269	444	713	58158	64592	42081	80669	122750
R43	315	360	268	407	675	56954	63148	44017	76085	120102
R44	336	349	266	419	685	56969	63262	43507	76724	120231
R45	338	325	259	404	663	56310	62460	41841	76929	118770
R46	316	342	263	395	658	56099	62175	43766	74508	118274
R47	329	374	261	442	703	59685	66199	41220	84664	125884
R48	349	343	261	431	692	57736	63839	41333	80242	121575
R49	326	344	263	407	670	57582	64162	42453	79291	121744

R50	340	346	275	411	686	57647	64358	42333	79672	122005
R51	367	367	274	460	734	59462	66422	40614	85270	125884
R52	333	340	265	408	673	58172	64407	42317	80262	122579
R53	366	352	284	434	718	57215	63982	42416	78781	121197
R54	343	349	260	432	692	58352	65020	42981	80391	123372
R55	352	354	283	423	706	58431	64938	42991	80378	123369
R56	351	379	272	458	730	58841	65885	42039	82687	124726
R57	353	346	252	447	699	58947	65809	38607	86149	124756
R58	341	354	270	425	695	57797	64209	43043	78963	122006
R59	341	344	259	426	685	57682	64049	42200	79531	121731
R60	332	316	274	374	648	55708	61405	43028	74085	117113
R61	330	345	258	417	675	56061	61439	42646	74854	117500
R62	358	352	263	447	710	58018	63976	41577	80417	121994
R63	348	378	266	460	726	60622	67262	40155	87729	127884
R64	340	376	253	463	716	59423	66066	41403	84086	125489
R65	349	366	262	453	715	60305	67004	40091	87218	127309
R66	352	363	255	460	715	59151	65661	41077	83735	124812
R67	330	359	254	435	689	60179	66733	42846	84066	126912
R68	332	348	256	424	680	59906	66487	42642	83751	126393
R69	359	359	253	465	718	60496	66740	40911	86325	127236
R70	366	382	253	495	748	61028	67984	40554	88458	129012
R71	328	349	263	414	677	60160	66702	43583	83279	126862
R72	342	382	268	456	724	58994	65574	41546	83022	124568
R73	334	347	264	417	681	57203	63498	43517	77184	120701
R74	331	339	273	397	670	55622	61223	43815	73030	116845
R75	313	360	264	409	673	56973	63238	43676	76535	120211
R76	329	352	242	439	681	59250	65228	41580	82898	124478
R77	326	367	271	422	693	59580	65955	41965	83570	125535
R78	345	379	271	453	724	59890	66669	41568	84991	126559
R79	345	363	271	437	708	58349	65187	42378	81158	123536
R80	343	347	277	413	690	57449	63642	43578	77513	121091
R81	346	344	269	421	690	58284	64896	42789	80391	123180
R82	351	350	267	434	701	58248	64764	42333	80679	123012
R83	356	367	276	447	723	59398	66226	41740	83884	125624
R84	342	339	272	409	681	56201	61906	42516	75591	118107
R85	323	320	258	385	643	57217	63384	42637	77964	120601
R86	343	361	261	443	704	59174	65871	41769	83276	125045
R87	337	335	286	386	672	56045	61947	42492	75500	117992
R88	330	356	269	417	686	57237	63173	42721	77689	120410
R89	332	341	260	413	673	58303	64581	41907	80977	122884
R90	320	338	262	396	658	58235	64510	41828	80917	122745
